# Research on Dynamic Measurement Method of Flow Rate in Tea Processing

**DOI:** 10.3390/s22114294

**Published:** 2022-06-05

**Authors:** Zhangfeng Zhao, Gaohong Liu, Yueliang Wang, Jiyu Peng, Xin Qiao, Jiang Zhong

**Affiliations:** 1 College of Mechanical Engineering, Zhejiang University of Technology, Hangzhou 310023, China; i12fly@163.com (Z.Z.); ghlzjut2022@163.com (G.L.); jypeng@zjut.edu.cn (J.P.); diana27qiao@zjut.edu.cn (X.Q.); 2 Key Laboratory of Special Purpose Equipment and Advanced Manufacturing Technology Ministry of Education, Zhejiang University of Technology, Hangzhou 310023, China; 3 Ningbo Yaojiangyuan Machinery Co., Ltd., Ningbo 315444, China; gri-2009@vip.163.com

**Keywords:** tea flow, dynamic measurement, variational empirical mode decomposition, wavelet threshold, denoising

## Abstract

Tea flow rate is a key indicator in tea production and processing. Due to the small real−time flow of tea leaves on the production line, the noise caused by the transmission system is greater than or close to the real signal of tea leaves. This issue may affect the dynamic measurement accuracy of tea flow. Therefore, a variational mode decomposition combined with a wavelet threshold (VMD−WT) denoising method is proposed to improve the accuracy of tea flow measurement. The denoising method of the tea flow signal based on VMD−WT is established, and the results are compared with WT, VMD, empirical mode decomposition (EMD), and empirical mode decomposition combined with wavelet threshold (EMD−WT). In addition, the dynamic measurement of different tea flow in tea processing is carried out. The result shows that the main noise of tea flow measurement comes from mechanical vibration. The VMD−WT method can effectively remove the noise in the tea dynamic weighing signal, and the denoising performance is better than WT, VMD, EMD, and EMD−WT methods. The average cumulative measurement accuracy of the tea flow signal based on the VMD−WT algorithm is 0.88%, which is 55% higher than that before denoising. This study provides an effective method for dynamic and accurate measurement of tea flow and offers technical support for digital control of the tea processing.

## 1. Introduction

Dynamic measurement of the flow rate during tea processing is an important part of the tea automatic production line, and the measurement accuracy is a key factor to ensure the tea quality [1]. The subsequent processing parameters can be adjusted by monitoring the flow rate of tea [2], such as tea fixation time, drying time, etc. An excessive tea flow rate will lead to uneven heating of tea and quality reduction [3]. An electronic belt scale is often used for tea flow rate dynamic measurement. During production, due to the small real−time tea flow, the weighing signal is affected by various noise sources, such as mechanical vibration, environmental changes, and electromagnetic interference, resulting in low dynamic measurement accuracy of the tea flow rate.

To improve the dynamic measurement accuracy of the tea flow rate, it is necessary to use signal processing methods to denoise the dynamic weighing signal of tea. Commonly used weighing signal denoising methods include the Kalman filter method, model−based identification method, adaptive filtering method, neural network, least squares support vector machine, decision regression tree, wavelet threshold denoising [4], and signal decomposition [5,6,7,8], etc. Wang et al. [9] proposed a Kalman filter combined with piecewise linear interpolation calibration to solve the problems of nonlinear calibration and precision improvement of electronic scales, but only external noise was suppressed. Dario et al. [10] proposed Shaper−Based Filters (SBFs) to ensure accurate, robust, and rapid estimation of the mass of the measurand. Pawlowski et al. [11] proposed adaptive filtering combined with a centrifugal force compensation (CFC) to improve weight estimation precision, especially at higher operating speeds. A BP neural network model was built by Li et al. [12] to compensate the auto−weighing error of the belt scale and greatly improved measurement accuracy. Jiang et al. [13] proposed a SVM based on an improved particle swarm optimization (NAPSO) to predict the dynamic measurement errors, which had a higher accuracy and a smaller prediction error. The decision regression tree was used to predict the measurement error by Wu et al. [14], which was simple, fast, and had a better prediction performance than least square estimation.

During dynamic measurement of tea flow, the noise mainly comes from mechanical vibration. These noises are non−stationary signals and overlap with the effective signal in the frequency spectrum, which cannot be removed using the above methods. The wavelet threshold denoising method offers advantages such as multi−resolution and decorrelation, and is suitable for denoising of non−stationary signals [15]. However, the selection of the wavelet basis function and decomposition level has a great influence on the denoising results and has certain limitations [16]. Empirical mode decomposition (EMD) is an adaptive denoising method. The signal is decomposed into several intrinsic mode functions (IMFs), and then the Hilbert–Huang transformation, spectrum analysis, processing, and reconstruction are carried out on the IMFs to realize the denoising of the signal [17]. However, EMD is prone to produce modal aliasing and endpoint effects when decomposing the signal, and the reconstructed signal will still have noise [18]. Although EMD−WT offers good adaptability and a reliable theoretical and mathematical derivation basis, in practical engineering applications, high−frequency interference components still exist in the signal spectrum [19]. The signal spectrum interval after EMD−WT decomposition is not accurate enough, and the fault feature information cannot be accurately extracted. Zhuang et al. [20] proposed a piecewise cubic Hermite interpolation polynomial combined with empirical wavelet transform algorithm (PCHIP−EWT) for noisy and non−stationary signal processing. The results showed that PCHIP−EWT could effectively separate components with a similar spectrum, and it was more suitable for analyzing noise and non−stationary signals than EWT. Sun et al. [21] proposed a new surface EMG signal denoising algorithm based on an ensemble empirical mode decomposition combined with a wavelet threshold (EEMD−WT), which demonstrated a good denoising effect under white noise interference. It could effectively remove the random noise in surface EMG signals, but it lacked stability.

Therefore, here, a novel denoising method on VMD−WT is proposed for dynamic and rapid detection of the tea flow rate during processing. The specific objectives are as follows: (1) study the dynamic weighing signal of tea leaves based on the spectrum analysis method, (2) investigate and establish the denoising method of the tea flow signal based on VMD−WT, and compare it to the traditional denoising methods (WT, EMD, VMD, and EMD−WT), and (3) apply the method to the actual tea flow measurement to verify the effectiveness of the method.

## 2. Materials and Methods

### 2.1. Sample Preparation

Tea leaves (Longzhu green tea, grade 4) were collected from a local producer in Dalan Town, with an average altitude above 550 m, between 121°04′31″~121°11′01″ E and 29°45′04″~29°51′47″ N. A conventional green tea processing method (including withering, first fixation, first rolling, second fixation, second rolling, and drying) was employed. It was carried out by a local tea processing factory (Ningbo Yaojiangyuan Machinery Co., Ltd., Ningbo, China).

### 2.2. Experimental Device

**Figure 1:**
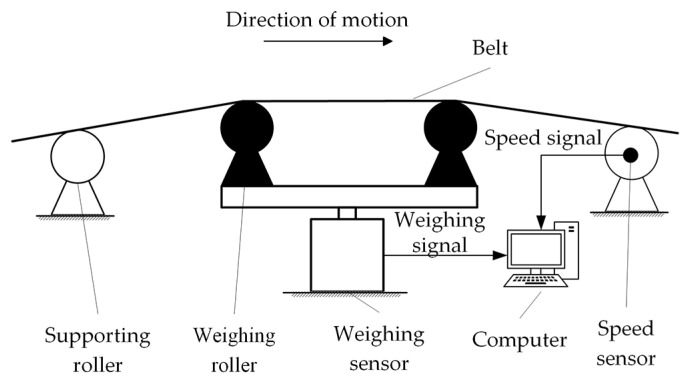
Schematic diagram of the experimental device.

Tea flow rate measurement was carried out in a self−developed electronic belt scale. Figure 1 is the schematic diagram of the experimental device. The structure of the electronic belt scale is a suspended carrier. The weighing sensor was placed under the weighing roller. The weight of the tea leaves was directly transferred to the weighing sensor via the weighing rollers. The weight signal (detected by the weighing sensor) and speed signal (detected by the speed sensor) were transmitted to the computer for further analysis.

### 2.3. Data Processing Method

#### 2.3.1. Principle of VMD

Variational mode decomposition (VMD) is a novel variable−scale adaptive decomposition method proposed by Dragomiretskiy et al. [22]. It can choose the decomposition level by itself and avoid modal aliasing and the endpoint effect, to an extent [23,24]. The VMD algorithm finds the optimal variational model through multiple iterations [25] and decomposes the complex original signal into multiple modal components, $u_{i}$, with sparse characteristics. Among them, each modal component could achieve the determined center frequency and frequency bandwidth through Hilbert–Huang transformation, and the sum of the frequency bandwidth of each component in the decomposition result is the smallest. The original signal can be obtained by adding each component. Thus, the constrained variational equation of the variational mode decomposition is:

(1) $$\begin{array}{l} \underset{{}_{\left\{u_{k}\},\{\omega_{k}\right\}}}{\min}\left\{\sum_{k}{\left\|\partial_{t}\left[\left(\delta \left(t\right)+j/\left(\pi t\right)\right)*u_{k}\left(t\right)\right]e^{-j\omega_{k}t}\right\|}_{2}^{2}\right\}\\ s.t.\sum_{k}u_{k}=f\left(t\right) \end{array}$$

where $u_{k}$ is the K−th modal component, $\omega_{k}$ is the center frequency of the K−th component, $\partial_{t}$ is the variation function with respect to time, δ(t) is the unit impulse function, ∗ is the convolution symbol, ${\left\|\cdot \right\|}_{2}^{2}$ is the square of the 2-norm of the vector, and $\underset{k}{\sum }u_{k}=f\left(t\right)$ is the constraint condition to ensure that the sum of all modal components is the original signal.

The quadratic penalty parameter, α, and the Lagrangian multiplier, λ, were introduced to turn finding the minimum value of the constrained problem into finding the local optimal solution of the unconstrained model. The augmented Lagrange function is shown in Formula (2):

(2) $$\begin{array}{l} ℒ\left(\left\{u_{k}\right\},\left\{\omega_{k}\right\},\lambda \right)=\alpha {\sum_{k}\left\|\partial_{t}\left[\left(\delta \left(t\right)+j/\pi t\right)*u_{k}\left(t\right)\right]e^{-j\omega_{k}t}\right\|}_{2}^{2}\\ +{\left\|f\left(t\right)-\sum_{k}u_{k}\left(t\right)\right\|}_{2}^{2}+\langle \lambda \left(t\right),f\left(t\right)-\sum_{k}u_{k}\left(t\right)\rangle \end{array}$$

where λ is the Lagrangian multiplier, and α is the quadratic penalty parameter.

The alternating direction method of multipliers (ADMM) was used to iteratively update $u_{k}^{n+1}$, $\omega_{k}^{n+1}$, and $\lambda^{n+1}$. The optimal solution of the constrained variational model can be obtained by searching the saddle point through Formula (2).

The following VMD iterative operation is as follows:

(1) Initialize $\{u_{k}^{1}\},\;\{\omega_{k}^{1}\},\;\lambda^{1},\;n=0$.
(2) The iterative $u_{k}^{n+1}\;\mathrm{and}\;\omega_{k}^{n+1}$ were transformed from time domain to frequency domain by using the Parseval/Plancherel Fourier equidistant method under the norm, and the following results were obtained, as shown in Formulas (3) and (4):
  (3) $${\hat{u}}_{k}^{n+1}\left(\omega \right)=\frac{\hat{f}\left(\omega \right)-\sum_{i\neq k}{\hat{u}}_{i}\left(\omega \right)+\hat{\lambda }\left(\omega \right)/2}{1+2\alpha {\left(\omega -\omega_{k}\right)}^{2}}$$
  (4) $${\hat{\omega }}_{k}^{n+1}=\frac{\int_{0}^{\infty }\omega {\left|{\hat{u}}_{k}\left(\omega \right)\right|}^{2}d\omega }{\int_{0}^{\infty }{\left|{\hat{u}}_{k}\left(\omega \right)\right|}^{2}d\omega }$$
  where ${\hat{u}}_{k}^{n+1}\left(\omega \right)$ is the Wiener filter of the current remainder, with a prior $1/{\left(\omega -\omega_{k}\right)}^{2}$, and the time domain is calculated as the real part of ${\hat{u}}_{k}^{n+1}\left(\omega \right)$ by Fourier transform, where ${\hat{\omega }}_{k}^{n+1}$ is the center frequencies of the modal component, and where $\hat{f}\left(\omega \right)$ is the frequency domain form of f(t).
(3) λ is updated as shown in Formula (5):
  (5) $${\hat{\lambda }}^{n+1}\left(\omega \right)={\hat{\lambda }}^{n}\left(\omega \right)+\tau \left(\hat{f}\left(\omega \right)-\sum_{k}{\hat{u}}_{k}^{n+1}\left(\omega \right)\right)$$
  where ${\hat{\lambda }}^{n+1}\left(\omega \right)$ is the Lagrange operator after iteration, and τ is the noise−tolerance parameter.
(4) The suspensive condition of iteration is shown in Formula (6):
  (6) $$\sum_{k}{\left\|{\hat{u}}_{k}^{n+1}-{\hat{u}}_{k}^{n}\right\|}_{2}^{2}/{\left\|{\hat{u}}_{k}^{n}\right\|}_{2}^{2}<\varepsilon$$
  where ε is convergence criterion. Step (2) is repeated until the function converges, which is to satisfy the condition of Formula (6).

#### 2.3.2. Principle of Wavelet Threshold Denoising

Wavelet transform is a time–frequency joint analysis method with variable resolution, which could automatically adjust the time window and frequency window according to the changing characteristics of the signal [26]. The wavelet coefficient of useful signals and noise interference were obtained by discrete wavelet transform of noisy signals [27]. According to the mathematical statistics of the two signals, the useful signal usually contains the low−frequency part with stable behavior and the high−frequency part with unstable change, and the larger amplitude is random signal, while the noise signal is usually the high−frequency part of the signal, and the smaller amplitude is dispersed in the periodic signal of the whole signal acquisition waveform [28]. Therefore, the respective wavelet coefficients after wavelet transform have different trends with the change of scale. The wavelet coefficients of useful signals increase or remain unchanged with the increase of scale, and the noise decreases with the increase of scale. Noise and useful signals can be effectively separated.

The basic principle of wavelet transform is to convolve the wavelet function, ψ(t), and the signal function, f(t). The signal is discrete. To facilitate processing, the continuous wavelet transform has to be discretized. The discrete wavelet basis function, ψ(t), is set. After scaling a and shifting b, $a=a_{0}^{j}$, $b=ka_{0}^{j}b_{0}$ can be taken to obtain a family of function as $\psi_{j,k}\left(t\right)$, as shown in Formula (7). The convolution of $\psi_{j,k}\left(t\right)$ and signal f(t) can obtain the discrete wavelet transform, $DWT_{f}\left(b,a\right)$, as shown in Formula (8), and the inverse transform was performed to obtain the reconfigurable signal f(t), as shown in Formula (9).

(7) $$\psi_{j,k}\left(t\right)=a_{0}^{-j/2}\psi \left(a_{0}^{-j}t-kb_{0}\right)$$

(8) $$DWT_{f}\left(b,a\right)=a^{-1/2}\int_{-\infty }^{+\infty }f\left(t\right)\psi^{*}\left(a_{0}^{-j}t-kb_{0}\right)dt$$

(9) $$f\left(t\right)=c\sum_{-\infty }^{+\infty }\sum_{-\infty }^{+\infty }d_{j,k}\psi_{j,k}\left(t\right)$$

where $d_{j,k}$ is the convolution f(t) and $\psi_{j,k}\left(t\right)$.

#### 2.3.3. Denoising Steps Based on VMD−WT Algorithm

When the VMD algorithm is used to denoise the dynamic weighing signal, the noisy signal is adaptively decomposed into multiple modal components, and the center frequency and bandwidth of each component are determined. However, the useful signal and the main noise in the signal are in the low−frequency part, and the wavelet threshold method has strong local time–frequency domain analysis ability. Therefore, we combined VMD and the wavelet threshold method, and applied it to the denoising of the tea dynamic measurement signal. The main steps of the algorithm are as follows:

(1) Decompose the noisy weighing signal into multiple IMFs through VMD. Selecting appropriate decomposition layers can effectively avoid over−decomposition or under−decomposition, which has a great influence on the decomposition result of the signal. Therefore, the instantaneous frequency mean method [29] was used to solve the decomposition level in this case. Hilbert–Huang transform was performed on IMFs to calculate the mean instantaneous frequency of each IMF component, and the mean instantaneous frequency at different decomposition levels, *K,* was compared. When there is a significant curvature change at a certain *K* value, the decomposition level is *K*−1.
(2) The modal component was determined to be signal−dominated or noise−dominated. The frequency domain analysis of the tea dynamic weighing signal was carried out to determine the frequency characteristics of the effective signal, and the modal components dominated by noise were removed according to the center frequency and bandwidth of each modal component. Then, the signal was reconstructed.
(3) The reconstructed signal was denoised by the wavelet threshold.
(4) The denoised signal was reconstructed by wavelet.

**Figure 2:**
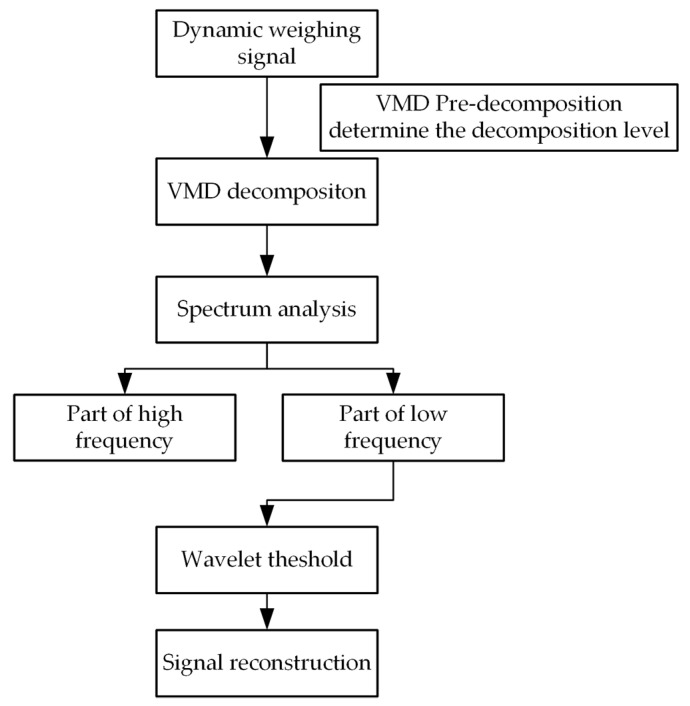
Flow chart of denoising of tea dynamic weighing signal based on VMD−WT.

The flow chart of denoising of the tea dynamic weighing signal based on VMD−WT is shown in Figure 2.

### 2.4. Evaluation Indicators

The signal−to−noise ratio (SNR) [30] was used to evaluate the denoising performance, which is calculated by Formula (10). The SNR is the ratio of useful signal power and noise power. The larger the SNR, the better the denoising performance. The weighing signal was converted into a weight value, and the measurement relative error was calculated. The relative error is calculated by Formula (11). The mean absolute error is the sum of all relative errors divided by the number of samples, as shown in Formula (12). The mean absolute error is usually used to represent the cumulative measurement accuracy of dynamic weighing devices.

(10) $$SNR=10\log_{10}\frac{P_{s}}{P_{n}}$$

(11) $$e=\left|q_{a}-q_{r}\right|/q_{r}\times 100\%$$

(12) $$\hat{e}=\frac{1}{n}\sum_{i=1}^{n}\left|e_{i}\right|$$

where $P_{s}$ is the power of useful signal, $P_{n}$ is the power of noise, e is the relative error, $q_{r}$ is the real weight, $q_{a}$ is the actual weight, and $\hat{e}$ is the mean absolute error.

## 3. Results and Analysis

### 3.1. Spectrum Analysis of Weighing Signal

The Fourier transform method could be used to analyze the frequency components contained in the weight signal, and extract features of the effective signal and noise. In practical operation, the non−load signal of the belt scale could be approximated as noise, and the loaded signal is subtracted from the non−load signal to obtain the approximate useful signal. Therefore, the Fourier transform method was used to analyze the spectrum of the non−load signal and the loaded signal, and to extract features of each component in the signal.

This experiment was carried out in the laboratory environment with a self−developed electronic belt scale. When the belt was stable during continuous measurement, the non−load signal and loaded signal of tea were collected from the weighing sensor, respectively. Signal acquisition experimental methodology was as follows: The non−load signal was measured at a speed of 0.2 m/s for a period time, followed by putting tea under the same working conditions, and keeping the flow rate at 1 kg/min. The acquired signal was divided into ten segments consisting of the same number of non−load signal points and loaded signal points, respectively. The sampling frequency of the signal was 1000 Hz, and the number of sampling points was 600.

**Figure 3:**
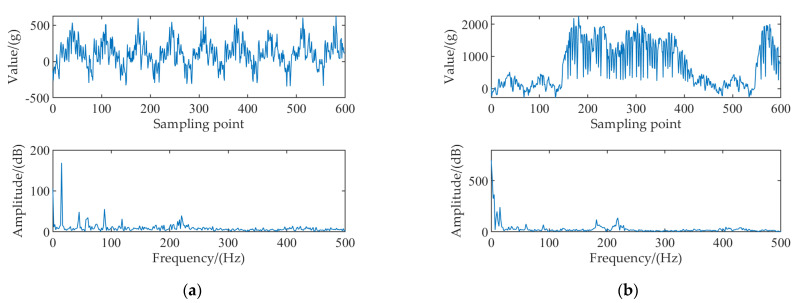
Spectrum analysis of the non−load and loaded signals of the electronic belt scale: (**a**) Non−load signal; (**b**) Loaded signal.

**Figure A1:**
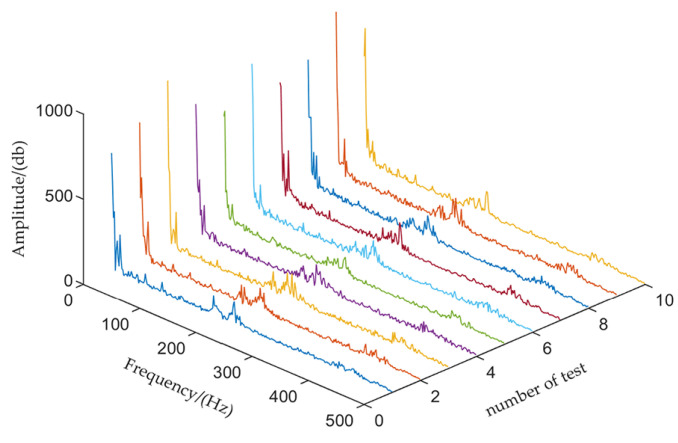
Spectrum diagram of non−load signal.

**Figure A2:**
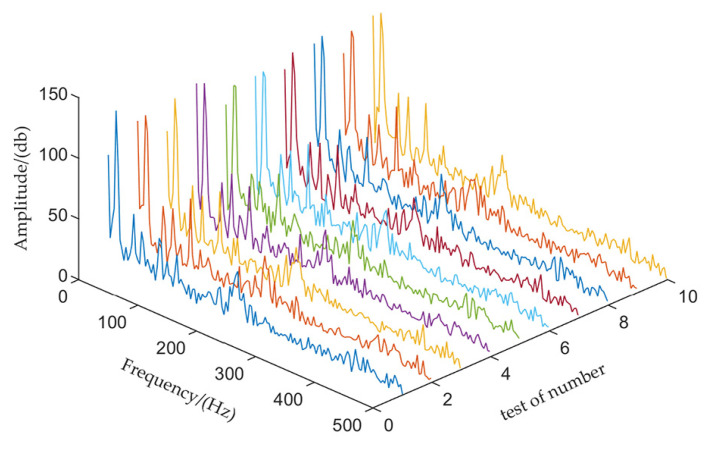
Spectrum diagram of loaded signal.

The collected signal was divided into ten sections, and the spectrum analysis was adapted. The results are shown in Appendix Figure A1 and Figure A2. The spectrum analysis of the non−load signal and loaded signal are shown in Figure 3a, b, respectively. The frequency component of the non−load signal was mainly located at 15 Hz, with a small number of frequency components at 45, 60, 90, 120, and 220 Hz. The frequency components of the loaded signal were located at 3.3 Hz, with a small number of frequency components at 10, 15, 45, 60, 90, 120, and 220 Hz.

During dynamic measurement of tea flow, there are mainly three kinds of interference signals: mechanical interference signal, material interference signal, and environmental interference signal. Among the above three interference signals, the mechanical interference signal is the main interference signal, which accounts for 50%. The material interference signal accounts for 40%, and its value decreases with the increase of weight. The environmental interference signal accounts for 10%, which is the minimum among three interference signals [31]. By comparing the spectrum statistical analysis of the non−load signal and the loaded signal, both contained a frequency component of 15 Hz, and its amplitude was about 200 dB. The loaded signal contained a frequency component of 10 Hz, with a slightly lower amplitude than that of the frequency component of 15 Hz. The mechanical interference signal was a 15 Hz frequency component, and the material interference signal was a frequency component of 10 Hz. In the load curve, the signal component with a frequency of about 3.3 Hz was the main component, while the noise signal was mainly the frequency component above 10 Hz, and mechanical interference signals, material interference signals, and weighing signals were in the low−frequency spectrum.

### 3.2. Time–Frequency Domain Analysis of Weighing Signal Based on VMD−WT

The VMD−WT method was further used to denoise the tea flow signal. The weighing signal was first decomposed by the VMD method, and the component that conformed to the frequency characteristics of the effective signal was extracted, and then the wavelet threshold was used to denoise the component to remove the noise with a similar frequency.

**Figure 4:**
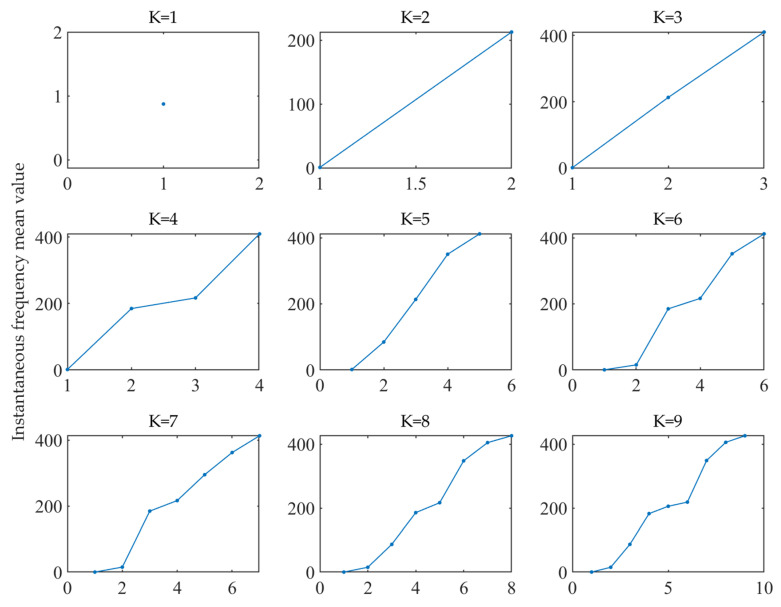
Instantaneous frequency mean value with different *K* values.

The decomposition layer number should be selected first before VMD analysis. According to the instantaneous frequency mean value method, the original signal was analyzed, and the decomposition level was selected here to be 1 to 9. Figure 4 shows the change of the corresponding instantaneous frequency mean value when different *K* values were used.

As shown in Figure 4, the instantaneous frequency mean value of each component presented obvious curve changes, when *K* was 6. Hence, level 5 was chosen as the decomposition level in this case. In other words, the signal was decomposed into 5 IMF components. The parameters of the VMD method were set as follows: decomposition level was 5, the penalty factor, α, was 2000, the convergence criterion, ε, was $10^{-7}$, and the initial center frequency, ω, was 0.

**Figure 5:**
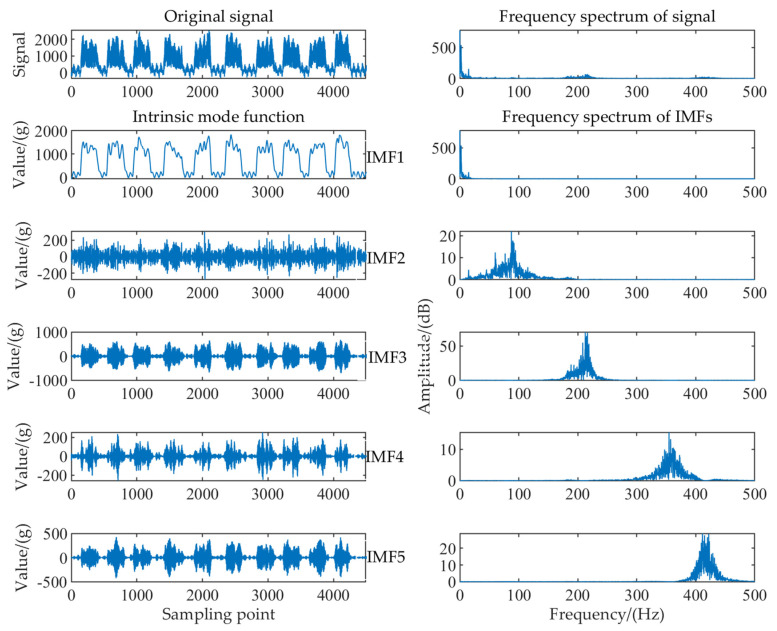
VMD decomposition results and spectrum analysis.

Figure 5 shows VMD decomposition results of the tea dynamic weighing signal. The main frequency of each IMF component was determined by the instantaneous frequency mean value method, as shown in Table 1. According to the spectrum analysis of the weighing signal in Figure 3, the effective components in the signal were mainly the low−frequency part below 10 Hz, namely the IMF1 component with the main frequency of 3 Hz. The IMF component with the main frequency greater than 10 Hz was removed. However, after reconstructing the IMF1 component, the SNR of the signal was not high, and the denoising performance was not ideal. According to the spectrum analysis of IMF1, a small number of frequency components around 10 and 15 Hz still existed, and the frequency of both the noise signal and the effective signal was in the low−frequency range. The VMD method could remove most of the high−frequency noise caused by environmental interference, while it was not able to effectively separate the noise signal from the IMF1. Therefore, further study should be carried out to remove the 10 and 15 Hz frequency components in the IMF1.

**Figure 6:**
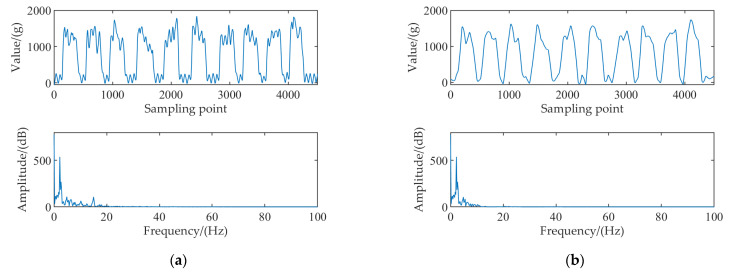
VMD and VMD−WT denoising results of the tea dynamic weighing signal: (**a**) VMD denoising result and spectrum analysis; (**b**) VMD−WT denoising result and spectrum analysis.

**Figure 7:**
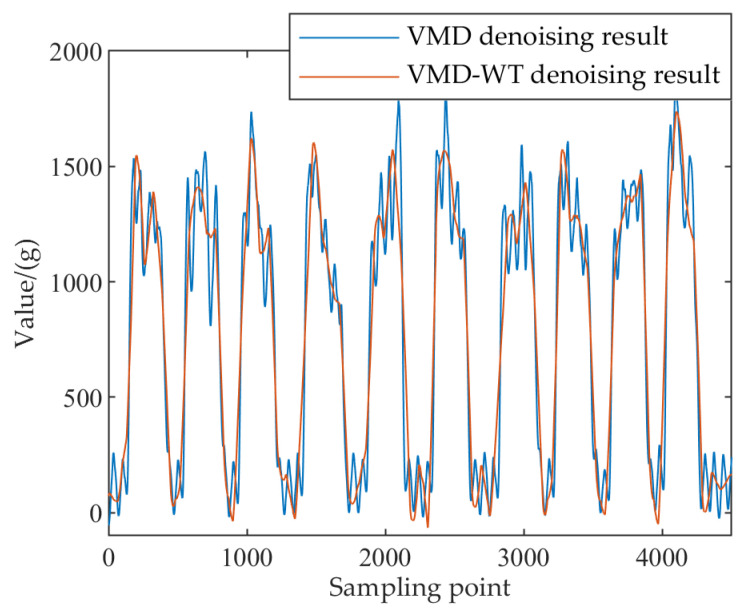
Comparison of VMD and VMD−WT denoising results of the tea dynamic weighing signal.

The WT method was performed after the reconstruction of the IMF1 component. The parameters of the WT method were as follows: the decomposition level was 6, the wavelet basis function was “sym6”, and the threshold function was set as hard thresholding. Figure 6 shows the VMD−WT and VMD denoising results of the tea dynamic weighing signal. The frequency components above 10 Hz were successfully removed after denoising with VMD−WT. As shown in Figure 7, the signal curve was smoother than the VMD reconstruction. This means that the mechanical interference noise and weighing object interference noise were removed, and the effective noise within 10 Hz was retained. It can be concluded that the VMD−WT method can effectively remove noise from the time–frequency domain weight signal.

### 3.3. Comparison of Denoising Results Based on Different Methods

To verify the denoising performance of VMD−WT, four traditional denoising methods (WT, EMD, EMD−WT, and VMD) were also used to remove the noise of the tea dynamic weighing signal, and the SNR was used to evaluate the denoising performance.

**Figure 8:**
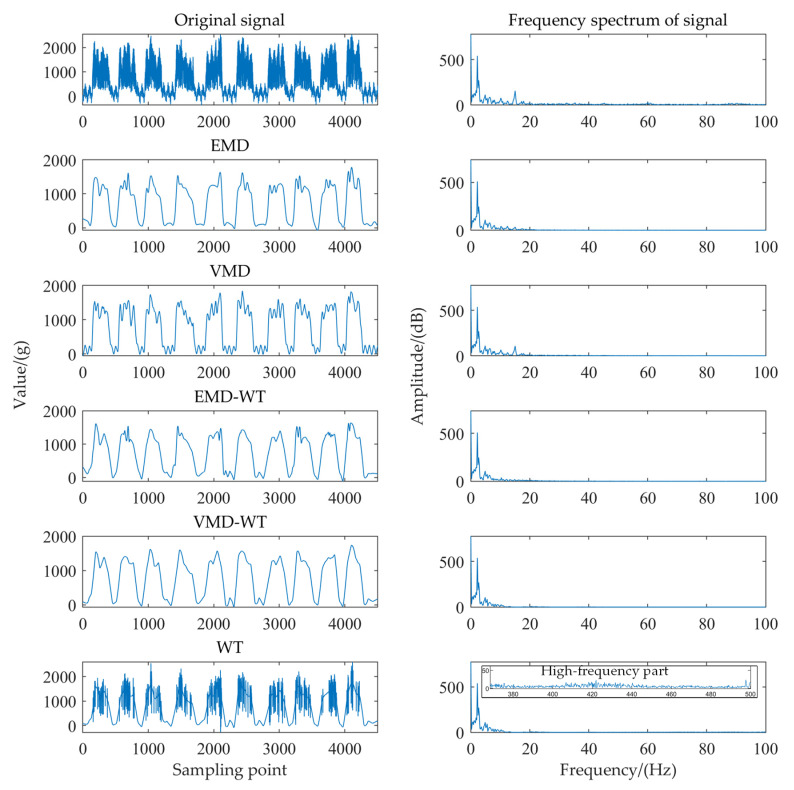
Frequency spectrum of the tea dynamic weighing signal with different denoising methods.

**Figure A3:**
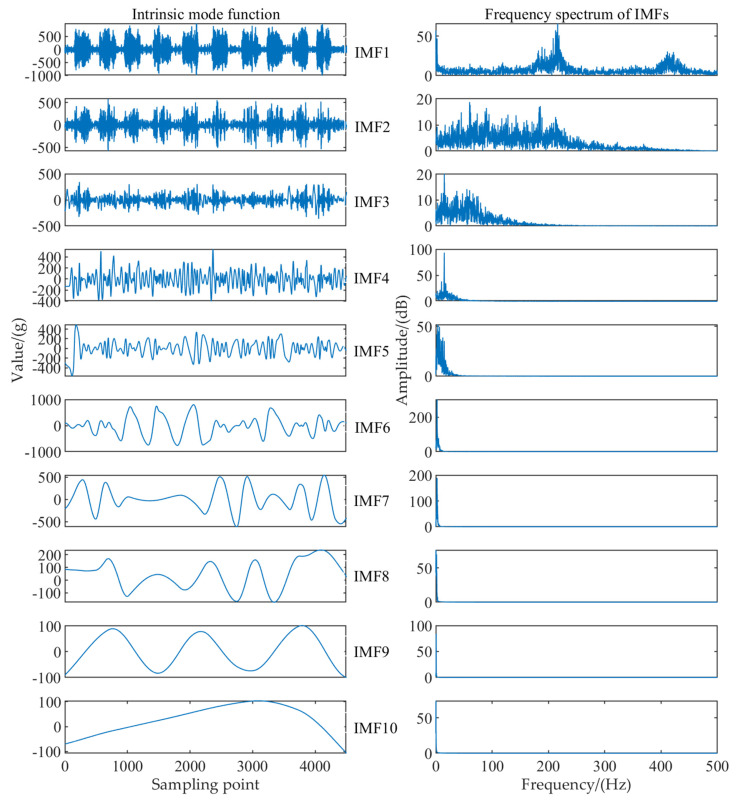
Decomposition result of the EMD method.

Figure 8 shows the frequency spectrum of the tea dynamic weighing signal with different denoising methods. The denoising performance of EMD was slightly better than that of VMD, while some of the useful signals were removed. The modal aliasing might exist during signal decomposition [32], as shown in Appendix Figure A3. It indicates that VMD performed better in signal feature extraction. The WT method was not effective in removing high−frequency noise, but it showed better denoising results in the low−frequency range. Moreover, the EMD method and the VMD method had better capability to denoise high−frequency noise. Both the EMD−WT method and the VMD−WT method showed good denoising capability for the tea dynamic weighing signal.

**Figure 9:**
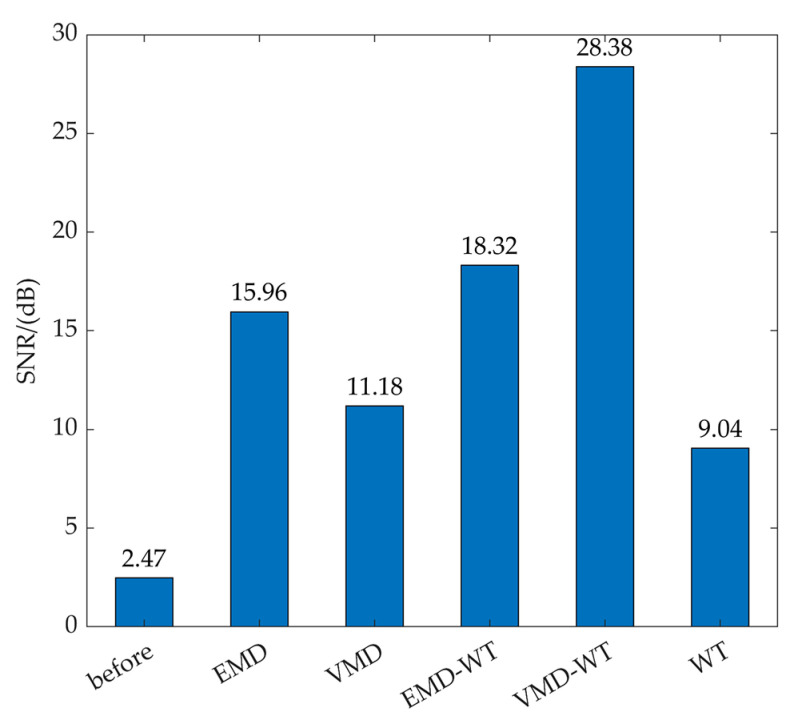
SNR of the tea dynamic weighing signal with different denoising methods.

Figure 9 shows the SNR of the tea dynamic weighing signal with different denoising methods. Among the five methods, the weighing signal denoising based on the VMD−WT method showed the best results, with a SNR of 28.38 dB. The measurement sensitivity and accuracy of tea flow were greatly improved compared with the raw signal (the SNR was 2.47 dB). Therefore, the VMD−WT method is an effective method for adaptively removing the noise in the tea weighing signal and improving the accuracy of the dynamic measurement of tea flow.

### 3.4. Dynamic Measurement Results of Tea Flow Based on VMD−WT

To further verify the effectiveness of this method, the weighing test was performed in the self−developed electronic belt scale, and the signals were collected ten times. The sampling frequency of the weighing system was 5 Hz, and the running speed of the belt scale was 0.2 m/s. To keep the test under the same working conditions, the electronic belt scale was kept continuously running. The experimental steps were as follows: First, the non−load experiments were carried out, and then the loaded experiments of 2000, 3000, 4000, and 5000 g of tea were carried out, respectively. Each group of weights was subjected to ten experiments, and the average error of each group of weights was calculated. The final cumulative error results are presented in Table 2.

As shown in Table 2, by counting the relative error of 50 measurements, the cumulative measurement error before denoising was 1.95%, and the cumulative measurement error after denoising was 0.88%. The cumulative measurement accuracy after denoising with the VMD−WT method was improved, which was 55% higher than the cumulative measurement accuracy before denoising. The experimental results show that the VMD−WT method could effectively remove the noise of the dynamic weighing signal of tea and improve the measurement accuracy in the dynamic measurement of the tea flow rate.

## 4. Conclusions

During the tea flow dynamic measurement, the noise caused by mechanical vibration and other factors disturbs the weighing signal, which leads to low measurement accuracy. A denoising method based on the VMD−WT method was proposed to adaptively remove the noise in the tea weighing signal and improve the accuracy of the dynamic measurement of tea. The VMD method was used to decompose the tea weighing signal, and the IMF components conforming to the frequency characteristics were extracted. Then, the WT method was used to remove the noise close to the effective signal frequency. The result showed that the noise was mainly mechanical interference noise (15 Hz) and weighing object interference noise (10 Hz). The VMD−WT method could effectively remove this part of the noise, and the denoising performance was better than that with WT, EMD, EMD−WT, and VMD. The cumulative measurement accuracy of tea flow measurements remained at about 0.88%, which was 55% higher than the measurement accuracy before denoising. This study provides a novel method for improving the dynamic measurement accuracy of the tea flow rate, which is important for tea digital control and processing.

## Figures and Tables

**Table 1:** The main frequency of each IMF component.

	IMF1	IMF2	IMF3	IMF4	IMF5
Main frequency	3 Hz	87 Hz	216 Hz	354 Hz	411 Hz

**Table 2:** Comparison of cumulative measurement error results after denoising and before denoising.

Test	2000 g		3000 g		4000 g		5000 g	
	before	after	before	after	before	after	before	after
No.1	2.38%	1.15%	1.84%	0.77%	1.50%	0.51%	1.29%	0.09%
No.2	1.98%	0.95%	1.64%	0.48%	2.56%	1.54%	1.22%	0.40%
No.3	2.57%	1.23%	1.21%	0.45%	1.48%	0.45%	1.31%	0.56%
No.4	1.19%	0.34%	1.57%	0.65%	1.87%	0.59%	3.43%	1.78%
No.5	1.85%	0.57%	1.65%	0.66%	1.65%	0.55%	1.35%	0.57%
No.6	3.56%	1.65%	2.54%	1.33%	2.35%	1.02%	1.75%	0.94%
No.7	2.35%	1.23%	1.35%	0.53%	1.78%	0.85%	1.86%	0.85%
No.8	1.57%	0.67%	2.73%	1.27%	3.23%	1.44%	2.76%	1.32%
No.9	1.39%	0.85%	2.07%	1.02%	1.98%	0.75%	2.13%	1.56%
No.10	1.67%	0.89%	1.85%	0.83%	1.71%	0.62%	1.96%	1.21%
Cumulative measurement error before denoising			1.95%		Cumulative measurement error after denoising		0.88%	

## Data Availability

Not applicable.
